# 低共熔溶剂改性反相液相色谱法用于天然产物分离

**DOI:** 10.3724/SP.J.1123.2026.04013

**Published:** 2026-08-08

**Authors:** Hui ZHANG, Huimin WU, Qun HE, Tianxue QIN, Suying GUO, Wenjing LI, Rong JIANG, Guangying SUN

**Affiliations:** 黄山学院，安徽 黄山 245021; Huangshan University，Huangshan 245021，China

**Keywords:** 低共熔溶剂, 反相制备液相色谱, 离子对色谱, 天然产物, deep eutectic solvent （DES）, preparative reversed-phase liquid chromatography, ion-pair chromatography, natural products

## Abstract

复杂天然产物在液相色谱分离过程中，高载样量与良好分离效果通常难以兼顾。为了解决该技术瓶颈，本工作以低共熔溶剂（deep eutectic solvents， DESs）为流动相添加剂，系统探究其对不同类型化合物在C18柱上色谱行为规律的影响，包括保留值、过载峰形及吸附容量，并分别验证其在黄连、金银花及山茱萸提取物中分离小檗碱、绿原酸、莫诺苷的应用潜力。结果表明，对于可离子化化合物，在流动相中添加DESs能起到压缩色谱峰宽和提高分辨率的效果。其中，对碱性化合物而言，这种提升主要来自DESs中的氢键受体如氯化胆碱对固定相残留硅羟基的屏蔽。而酸性化合物分离的改善则取决于离子对的形成，这与流动相的pH及缓冲盐浓度高度相关。过载和吸附行为实验结果还表明，DESs能有效减弱酸性化合物在固定相吸附过程中溶质-溶质相互作用。对天然产物中的中性化合物而言，在流动相中添加DESs可通过调控离子型共洗脱杂质的色谱行为，间接实现分离优化。本研究为DESs作为流动相添加剂在天然产物制备分离优化中的应用提供了理论依据与技术参考。

## Abstract (en)

The composition of natural products is usually very complex. During the separation of natural products， high loading capacity and satisfactory chromatographic resolution are often hardly achieved. To address this limitation， various modifiers were added in the mobile phase to alter the chromatographic behavior. Here， deep eutectic solvents （DESs） were chosen to accomplish this objective on C18 column. Changes in retention factor， overloaded peak shape and adsorption capacity were systematically investigated and interpreted. The application potential of DESs in the separation of natural products was then evaluated. For ionizable compounds， the addition of DESs could compress the peak width and enhance the resolution. This is closely related to the electrostatic interactions provided by hydrogen bond acceptor （HBA） of DESs. For ionizable basic compounds like nortriptyline hydrochloride， they could remain in a cationic state constantly. Consequently， ion-exchange interactions occurred between nortriptyline cation and residual silanol anion， which resulted in severe peak tailing. However， this interaction could be shielded by the addition of HBA of DESs （such as choline chloride （ChCl））. As the pH of the mobile phase increased， the dissociation of silanol groups was enhanced. This rendered the shielding effect more prominent and led to a higher peak compression of nortriptyline hydrochloride. Notably， although the mobile phase was acidic， the dissociation of silanol groups cannot be completely suppressed. It means that this modulating effect of HBA never disappears. Based on these principles， the semi-preparative separation of berberine from *Coptis chinensis* extracts was successfully improved. The improvement in the separation of acidic compounds by DESs depends on the ion-pair formation. This effect is highly correlated with the mobile phase pH and salt concentration. Here， benzoic acid was chosen as a model to investigate the overload behavior and adsorption. The results indicated that under neutral conditions， ChCl addition converted the sigmoidal adsorption to a linear type. It can be attributed to the ion-pair formation between benzoate anions and HBA cations. Mutual repulsion between benzoate anions was therefore reduced and adsorption capacity correspondingly enhanced. Furthermore， the results of the overload experiment indicated that ChCl can effectively weaken the solute-solute interactions. It is worth noting that the mobile phase pH is barely influenced after the addition of ChCl. Remarkably， these features are of critical importance for the optimization of preparative chromatographic methods. The beneficial effect of HBAs was validated in the semi-preparative separation of chlorogenic acid from *Lonicera japonica* extracts. For neutral compounds like morroniside， the HBA in DESs cannot affect their chromatographic behavior through electrostatic interactions. However， there are usually many ionizable impurities surrounding the neutral compounds in natural products. Their chromatographic behavior can be modulated by the HBAs in DESs. The separation of neutral compounds in natural products could be indirectly optimized by modulating their chromatographic behavior. This view was also confirmed by the separation of morroniside from *Cornus officinalis* extracts.

反相液相色谱是天然产物分离中应用最广泛的技术手段之一。天然产物的组分复杂，仅依靠单一反相色谱模式难以实现化合物纯化。目前提升分离效能的常用策略主要分为3类：一是采用细粒径填充色谱柱或增加柱长，二是通过固定相改性^［1，2］^，三是优化流动相组成。其中，优化流动相组成（如添加离液剂如高氯酸钠、六氟磷酸钾）操作简单，效果显著，已成为色谱领域改善复杂样品分离性能的常用手段^［3-5］^。因此，开发绿色、经济且调控能力强的流动相添加剂，对解决天然产物分离中基质干扰问题具有重要现实意义。

低共熔溶剂（deep eutectic solvents， DESs）是由氢键受体（hydrogen bond acceptor， HBA）与氢键给体（hydrogen bond donor， HBD）相互作用产生的一种经济环保、挥发性低的新型绿色溶剂。目前在材料合成、样品前处理、萃取分离等领域展现出良好的应用前景^［6］^。近年来的研究表明，DESs作为色谱添加剂可有效调控色谱分离行为、优化峰形与分离选择性^［7］^。Li等^［8］^采用氯化胆碱（ChCl）-甘油型DESs作为流动相添加剂，显著提升了咖啡酸在C18柱上的分离效果。Tan等^［9］^系统研究了DESs对生物碱类化合物在C18柱上分离的影响机制，证实DESs可通过对硅羟基效应与洗脱效应的调控改善分离效果。该团队^［10］^进一步验证了DESs调控亲水作用色谱分离6种碱基与核酸的可行性。Marchel等^［11］^则基于盐酸L-脯氨酸-木糖醇构建了DESs，经其调控后硅胶柱对极性和大体积芳香化合物展现出了特殊选择性。此外，DESs在超临界流体色谱、毛细管电泳、胶束液相色谱等分离方法中均表现出调控潜力^［12-14］^。然而，现有研究多集中于分析领域，针对DESs调控反相制备液相色谱分离天然产物机制的系统性研究仍较为匮乏。

基于此，本工作首先系统探究了DESs对酸、碱、中性化合物在C18反相制备色谱柱上保留与过载行为的影响，并深入阐释调控机理。之后选取黄连、金银花、山茱萸3种典型天然产物提取物为研究载体，采用反相半制备C18柱对其中的小檗碱、绿原酸与莫诺苷展开纯化验证，以期为DESs在天然产物分离中的应用研究提供实践参考。

## 1 实验部分

### 1.1 主要仪器、试剂与材料

S6000高效液相色谱仪（配有二极管阵列检测器，华谱科仪（北京）科技有限公司，中国）；PHS-3E pH计（上海仪电科学仪器股份有限公司，中国）；Easy SUPER SERIES超纯水机（力康（Heal Force）公司，中国）。一次性有机针筒过滤器（孔径0.45 μm）购自上海兴亚净化材料厂。

氯化胆碱（AR，98%，上海阿拉丁生化科技股份有限公司）；磷酸氢二钾（AR，98%）和甘油（AR，99%）（上海玻尔化学试剂有限公司）；磷酸（AR，含量≥85.0%）、氢氧化钾（AR，含量≥85.0%）和氯化钾（KCl，AR，含量≥99.5%）（西陇科学股份有限公司）；苯甲酸（AR，含量≥99.5%）、乙二醇（AR，含量≥99.0%）、柠檬酸（AR，含量≥99.5%）（国药集团化学试剂有限公司）；尿素（AR，含量≥99.0%，天津市大茂化学试剂厂）；四甲基氯化铵（AR，纯度98%）和四乙基氯化铵（AR，纯度98%）（天津希恩思生化科技有限公司）；乙腈（acetonitrile， ACN）和甲醇（HPLC级，纯度≥99.9%，湖北弗顿科学技术有限公司）。绿原酸（纯度98%）及金银花提取物（绿原酸含量10%，批号：SQJYH20250825）购自西安圣青生物科技有限公司，盐酸小檗碱（纯度98%，批号：C14283974）购自上海麦克林生化科技股份有限公司，黄连提取物（小檗碱含量50%，生产日期：20251017）购自河南洲科水处理设备有限公司，盐酸去甲替林（纯度98%，批号：1+N89400MIK1）购自天津希恩思生化科技有限公司，莫诺苷（HPLC级，纯度≥98%，批号：C17908112）购自上海麦克林生化科技股份有限公司，山茱萸提取物（批号：240814SZY）购自南京普怡生物科技有限公司。ACCHROM C18（10 μm）填料购自华谱科仪（大连）科技有限公司。

### 1.2 DESs的制备

本研究参照文献［^9^］，共制备了5种DESs。将所有组分按指定的物质的量之比混合后，置于80 ℃油浴中加热搅拌至形成清亮透明溶液，冷却后备用。5种DESs的组成及制备配比如表1所示。

**表1:** 5种DESs的组成

DES	Hydrogen bond acceptor （HBA）	Hydrogen bond donor （HBD）	Molar ratio of HBA to HBD	Preparation amounts/g
DES-A	choline chloride （ChCl）	urea	1∶2	11.17+9.60
DES-B	tetramethylammonium chloride	ethylene glycol	1∶3	8.77+14.90
DES-C	ChCl	citric acid	1∶1	11.17+15.37
DES-D	ChCl	glycerol	1∶2	11.17+14.73
DES-E	tetraethylammonium chloride	ethylene glycol	1∶3	13.26+14.90

### 1.3 分析型及半制备C18柱装填

通过匀浆法分别将C18填料装填在规格为250 mm×4.6 mm（分析型C18柱）和250 mm×10 mm（半制备型C18柱）的不锈钢柱管内。装填前，称取4 g和18.8 g填料，分别悬浮在10倍体积的匀浆液中，超声2 min后装填。装填压力为60 MPa，匀浆液与顶替液分别为四氯化碳-二氧六环（2∶1，体积比）和甲醇与异丙醇（1∶1，体积比）。

### 1.4 溶液配制

磷酸缓冲液（PB）的配制：通过KOH溶液中和H_3_PO_4_溶液的方式配制。具体方式如下（以pH 2.5，浓度为25.4 mmol/L的磷酸缓冲液的配制为例）：称取2.30 g磷酸（含量≥85.0%），转移至500 mL容量瓶，加水定容至刻度线，得到浓度为40 mmol/L的H_3_PO_4_溶液。再称取1.32 g KOH（含量≥85.0%），加少量水溶解后转移至500 mL容量瓶中，加水定容至刻度线，得到浓度为40 mmol/L的KOH溶液。将40 mmol/L的KOH溶液缓慢滴加至H_3_PO_4_溶液中并持续搅拌，使用pH计实时监测溶液pH，直至其达到预期pH 2.5。记录初始H_3_PO_4_溶液（70 mL）及经滴定后所得PB体积（110 mL），并根据物质的量守恒来计算PB的浓度。其他浓度及pH的PB采用类似方式配制。具体见支持信息（https://www.chrom-China.com）。

含有添加剂的PB的配制（以pH 2.5、 25.4 mmol/L PB及添加剂ChCl的浓度为60 mmol/L为例）：按上述步骤配制PB。称取8.38 g ChCl，加所配制的PB溶解后，转移至1 L容量瓶中并定容，得到ChCl添加浓度为60 mmol/L的PB。

盐酸去甲替林溶液：分别称取1 mg和10 mg盐酸去甲替林，加水溶解定容至10 mL，配制质量浓度为0.1 mg/mL和1.0 mg/mL的盐酸去甲替林溶液。

苯甲酸溶液：以甲醇-水（50∶50，体积比）为溶剂，分别配制质量浓度为0.042、0.25、1、5及10 mg/mL的苯甲酸溶液。

苯甲酸钾溶液：称取100 mg苯甲酸、54 mg KOH，加水溶解定容至10 mL，配制质量浓度为13.1 mg/mL的苯甲酸钾溶液。用水稀释，分别得到质量浓度为13.118、6.559、3.936、2.624、1.312、0.656、0.328、0.164、0.054 mg/mL的苯甲酸钾溶液。

盐酸小檗碱溶液：称取10 mg盐酸小檗碱，加水溶解定容至10 mL容量瓶中，配制质量浓度为1 mg/mL的盐酸小檗碱溶液。

莫诺苷溶液：称取20 mg莫诺苷，加甲醇-9.2 mmol/L PB （pH 6.5）（25∶75，体积比）溶解至2 mL，配制质量浓度10 mg/mL的莫诺苷溶液。

绿原酸钾溶液：称取绿原酸10 mg及1.86 mg KOH，加水溶解定容至10 mL，配制成质量浓度为1.11 mg/mL的绿原酸钾溶液。

### 1.5 苯甲酸吸附等温线的测定

苯甲酸溶液的配制（以pH 6.5、18.2 mmol/L的PB（添加60 mmol/L ChCl）为例）：量取950 mL含ChCl的PB与50 mL乙腈混合，得到溶液A；分别称取0.05 g和0.5 g苯甲酸，以溶液A为溶剂溶解并定容至1 L，配制0.05 mg/mL和0.5 mg/mL的苯甲酸溶液（分别作为溶液B）。

前沿色谱分析的流动相：先后以质量浓度为0.05 mg/mL和0.5 mg/mL的溶液B，通过高效液相色谱仪二元泵按体积分数10%、20%、30%、40%、50%、60%、70%、80%、90%、100%与溶液A在线混合，得到质量浓度范围为0.005~0.5 mg/mL的系列苯甲酸溶液，作为前沿色谱分析的流动相。

采用前沿色谱法测定苯甲酸在分析型C18柱上的吸附等温线^［15］^，色谱柱温度：25 ℃；扫描波长范围：200~400 nm。

以溶液A平衡分析型C18柱至基线稳定，将0.005 mg/mL的苯甲酸溶液以0.8 mL/min的流速通过色谱柱至流出液紫外吸收值达到一个平台。随后，将苯甲酸浓度更换至下一个浓度点，继续通过色谱柱直至流出液吸收值达到下一个平台。依次类推，直到达到最高浓度（苯甲酸质量浓度0.5 mg/mL），形成特征突破曲线。根据该曲线，可得到各质量浓度下苯甲酸在C18柱上的突破体积*V*_r_（即拐点处流动相的总量）。

色谱柱吸附量计算公式为*Q_i_* =（*C_i_* -*C_i_*_-1_）（*V*_r，_ *_i_* -*V*_0_）/*m*_ads_+*Q_i_*_-1_，其中，*Q_i_* 和*Q_i_*_-1_分别为第*i*和第*i*-1平台下的吸附量；*C_i_* 和*C_i_*_-1_为对应梯度混合流动相中苯甲酸的浓度；*V*_r，_ *_i_* 为第*i*个平台突破体积；*m*_ads_为分析型C18柱中填料的质量；*V*_0_为死体积（通过尿嘧啶标定）。

### 1.6 在分析型C18柱上的分离条件

盐酸去甲替林、苯甲酸、盐酸小檗碱、苯甲酸钾、绿原酸钾和莫诺苷的配制溶剂、溶液含量、进样体积、流动相、检测波长见表2。色谱柱温均为25 ℃；流速为1.0 mL/min。

**表2:** 各化合物在分析型C18柱上的分离条件

No.	Compound	Contents/（mg/mL）	Injection volumes/μL	Solvent for working solution	Mobile phase composition	Detection wavelength/nm
Determination of the effect of DESs on the retention factors of nortriptyline HCl and benzoic acid on the analytical column						
1	nortriptyline HCl	0.1	5	H_2_O	ACN-13.8 mmol/L phosphate buffer （PB， pH 2.5， *C*_DES-A_=20.78 mg/mL） （40∶60. All such ratios are volume ratios， and the same applies hereafter.）	230
2	nortriptyline HCl	0.1	5	H_2_O	ACN-13.8 mmol/L PB （pH 2.5， *C*_DES-B_=23.66 mg/mL） （40∶60）	230
3	nortriptyline HCl	0.1	5	H_2_O	ACN-13.8 mmol/L PB （pH 2.5， *C*_DES-C_=26.54 mg/mL） （40∶60）	230
4	nortriptyline HCl	0.1	5	H_2_O	ACN-13.8 mmol/L PB （pH 2.5， *C*_DES-D_=25.90 mg/mL） （40∶60）	230
5	nortriptyline HCl	0.1	5	H_2_O	ACN-13.8 mmol/L PB （pH 2.5， *C*_DES-E_=28.15 mg/mL） （40∶60）	230
6	nortriptyline HCl	0.1	5	H_2_O	ACN-13.8 mmol/L PB （pH 2.5， *C*_ChCl_=11.17 mg/mL） （40∶60）	230
7	benzoic acid	0.042	50	methanol-H_2_O （50∶50）	ACN-9.2 mmol/L PB （pH 6.5， *C*_DES-A_=20.78 mg/mL） （5∶95）	230
8	benzoic acid	0.042	50	methanol-H_2_O （50∶50）	ACN-9.2 mmol/L PB （pH 6.5， *C*_DES-B_=23.66 mg/mL） （5∶95）	230
9	benzoic acid	0.042	50	methanol-H_2_O （50∶50）	ACN-9.2 mmol/L PB （pH 6.5， *C*_DES-C_=26.54 mg/mL） （5∶95）	230
10	benzoic acid	0.042	50	methanol-H_2_O （50∶50）	ACN-9.2 mmol/L PB （pH 6.5， *C*_DES-D_=25.90 mg/mL） （5∶95）	230
11	benzoic acid	0.042	50	methanol-H_2_O （50∶50）	ACN-9.2 mmol/L PB （pH 6.5， *C*_DES-E_=28.15 mg/mL） （5∶95）	230
12	benzoic acid	0.042	50	methanol-H_2_O （50∶50）	ACN-9.2 mmol/L PB （pH 6.5， *C*_ChCl_=11.17 mg/mL） （5∶95）	230
Investigation of the effect of ChCl on the retention factor of nortriptyline HCl at different pH						
13	nortriptyline HCl	0.1	5	H_2_O	ACN-25.4 mmol/L PB （pH 2.5） （40∶60）	230
14	nortriptyline HCl	0.1	5	H_2_O	ACN-25.4 mmol/L PB （pH 2.5， *c*_ChCl_=60 mmol/L） （40∶60）	230
15	nortriptyline HCl	0.1	5	H_2_O	ACN-20.3 mmol/L PB （pH 4.2） （40∶60）	230
16	nortriptyline HCl	0.1	5	H_2_O	ACN-20.3 mmol/L PB （pH 4.2， *c*_ChCl_=60 mmol/L） （40∶60）	230
17	nortriptyline HCl	0.1	5	H_2_O	ACN-20.0 mmol/L PB （pH 5.5） （40∶60）	230
18	nortriptyline HCl	0.1	5	H_2_O	ACN-20.0 mmol/L PB （pH 5.5， *c*_ChCl_=60 mmol/L） （40∶60）	230
19	nortriptyline HCl	0.1	5	H_2_O	ACN-18.2 mmol/L PB （pH 6.5） （40∶60）	230
20	nortriptyline HCl	0.1	5	H_2_O	ACN-18.2 mmol/L PB （pH 6.5， *c*_ChCl_=60 mmol/L） （40∶60）	230
Investigation of the effect of ChCl or KCl concentration on the overloading behavior of nortriptyline HCl on analytical column						
21	nortriptyline HCl	1	100	H_2_O	ACN-13.8 mmol/L PB （pH 2.5， *c*_ChCl_=0-80 mmol/L） （40∶60）	290
22	nortriptyline HCl	1	100	H_2_O	ACN-13.8 mmol/L PB （pH 2.5， *c*_KCl_=0-80 mmol/L） （40∶60）	290
Investigation of the effect of DES-A， DES-C， and DES-D on the overloading behavior of nortriptyline HCl on analytical column						
23	nortriptyline HCl	1	100	H_2_O	ACN-13.8 mmol/L PB （pH 2.5， *C*_DES-A_=20.78 mg/mL） （40∶60）	290
24	nortriptyline HCl	1	100	H_2_O	ACN-13.8 mmol/L PB （pH 2.5， *C*_DES-C_=26.54 mg/mL） （40∶60）	290
25	nortriptyline HCl	1	100	H_2_O	ACN-13.8 mmol/L PB （pH 2.5， *C*_DES-D_=25.90 mg/mL） （40∶60）	290
Investigation of the effect of ChCl on the chromatographic behavior of berberine HCl on analytical column						
26	berberine HCl	1	5， 20， 40， 60， 100	H_2_O	ACN-13.8 mmol/L PB （pH 2.5） （35∶65）	290
27	berberine HCl	1	5， 20， 40， 60， 100	H_2_O	ACN-13.8 mmol/L PB （pH 2.5， *c*_ChCl_=80 mmol/L） （35∶65）	290
28	berberine HCl	1	5， 20， 40， 60， 100	H_2_O	ACN-9.2 mmol/L PB （pH 6.5） （35∶65）	290
Investigation of the effect of ChCl addition on the overloading behavior of benzoic acid on analytical column under different pH						
29	benzoic acid	5	50	methanol-H_2_O （50∶50）	ACN-40.0 mmol/L H_3_PO_4_ （pH 2.0） （34∶66）	275
30	benzoic acid	5	50	methanol-H_2_O （50∶50）	ACN-40.0 mmol/L H_3_PO_4_ （pH 2.0， *c*_ChCl_=60 mmol/L ） （34∶66）	275
31	benzoic acid	5	50	methanol-H_2_O （50∶50）	ACN-20.3 mmol/L PB （pH 4.2） （29∶71）	275
32	benzoic acid	5	50	methanol-H_2_O （50∶50）	ACN-20.3 mmol/L PB （pH 4.2， *c*_ChCl_=60 mmol/L） （29∶71）	275
33	benzoic acid	5	50	methanol-H_2_O （50∶50）	ACN-20.0 mmol/L PB （pH 5.5） （11.5∶88.5）	275
34	benzoic acid	5	50	methanol-H_2_O （50∶50）	ACN-20.0 mmol/L PB （pH 5.5， *c*_ChCl_=60 mmol/L） （11.5∶88.5）	275
35	benzoic acid	5	50	methanol-H_2_O （50∶50）	ACN-18.2 mmol/L PB （pH 6.5） （5∶95）	275
36	benzoic acid	5	50	methanol-H_2_O （50∶50）	ACN-18.2 mmol/L PB （pH 6.5， *c*_ChCl_=60 mmol/L） （5∶95）	275
Investigation of the effect of ChCl or KCl on the overloading behavior of benzoic acid on analytical column						
37	benzoic acid	0.042， 0.25， 1， 10	50	methanol-H_2_O （50∶50）	ACN- 9.2 mmol/L PB （pH 6.5） （5∶95）	290
38	benzoic acid	0.042， 0.25， 1， 10	50	methanol-H_2_O （50∶50）	ACN- 9.2 mmol/L PB （pH 6.5， *c*_ChCl_=80 mmol/L） （5∶95）	290
39	benzoic acid	0.042， 0.25， 1， 10	50	methanol-H_2_O （50∶50）	ACN- 9.2 mmol/L PB （pH 6.5， *c*_KCl_=80 mmol/L） （5∶95）	290
Investigation of the effect of ChCl or KCl on the overloading behavior of potassium benzoate on analytical column						
40	potassium benzoate	13.118，6.559，3.936，2.624，1.312，0.656，0.328，0.164，0.054	50	H_2_O	ACN-8.1 mmol/L PB （pH 7.0） （5∶95）	270
41	potassium benzoate	13.118，6.559，3.936，2.624，1.312，0.656，0.328，0.164，0.054	50	H_2_O	ACN-8.1 mmol/L PB （pH 7.0， *c*_KCl_=80 mmol/L） （5∶95）	270
42	potassium benzoate	13.118，6.559，3.936，2.624，1.312，0.656，0.328，0.164，0.054	50	H_2_O	ACN-8.1 mmol/L PB （pH 7.0， *c*_ChCl_=80 mmol/L） （5∶95）	270
Investigation of the effect of ChCl on the overloading behavior of potassium chlorogenate on analytical column						
43	potassium chlorogenate	1.11	5， 20， 40， 60， 100	H_2_O	ACN-9.2 mmol/L PB （pH 6.5） （5∶95）	370
44	potassium chlorogenate	1.11	5， 20， 40， 60， 100	H_2_O	ACN-9.2 mmol/L PB （pH 6.5， *c*_ChCl_=80 mmol/L） （5∶95）	370
Investigation of the effect of ChCl on the overloading behavior of morroniside on analytical column						
45	morroniside	10	40， 80， 100	methanol-9.2 mmol/L PB （pH 6.5） （25∶75）	methanol-9.2 mmol/L PB （pH 6.5） （25∶75）	270
46	morroniside	10	40， 80， 100	methanol-9.2 mmol/L PB （pH 6.5） （25∶75）	methanol-9.2 mmol/L PB （pH 6.5， *c*_ChCl_=80 mmol/L） （25∶75）	270

### 1.7 黄连、金银花、山茱萸提取物的半制备分离条件

提取物的样品配制溶剂、浓度、进样体积、流动相、检测波长见表3。色谱柱温均为25 ℃；流速为2.5 mL/min。所有提取物溶液均用一次性针筒式过滤器过滤后再进样分析。

**表3:** 各植物提取物在半制备型C18柱上的分离条件

No.	Sample	Content/（mg/mL）	Injection volume/μL	Solvent for working solution	Mobile phase composition	Detection wavelengths/nm
1	*Coptis chinensis*extracts	100	100	methanol-H_2_O （50∶50）	ACN-9.2 mmol/L PB （pH 6.5） （35∶65）	290
2	*Coptis chinensis*extracts	100	100	methanol-H_2_O （50∶50）	ACN-9.2 mmol/L PB （pH 6.5， *c*_ChCl_=80 mmol/L） （35∶65）	290
3	*Lonicera Japonica* extracts	100	50	aqueous KOH solution	ACN-9.2 mmol/L PB （pH 6.5） （5∶95）	350
4	*Lonicera Japonica* extracts	100	50	aqueous KOH solution	ACN-9.2 mmol/L PB （pH 6.5， *c*_ChCl_=80 mmol/L） （5∶95）	350
5	*Cornus officinalis* extracts	100	100	mobile phase	methanol-9.2 mmol/L PB （pH 6.5） （25∶75）	254/270
6	*Cornus officinalis* extracts	100	100	mobile phase	methanol-9.2 mmol/L PB （pH 6.5， *c*_ChCl_=80 mmol/L） （25∶75）	254/270

## 2 结果与讨论

### 2.1 DESs作为添加剂对C18柱上酸碱保留行为的调控

在流动相中添加DESs对可离子化酸、碱化合物在C18柱上保留行为的调控作用存在明显差异。对可离子化碱性化合物而言，其在反相固定相常用pH范围（2~8）内通常保持离子化状态。DESs对其色谱行为的调控，通常与碱性化合物阳离子和硅羟基之间的静电相互作用有关。以盐酸去甲替林为例，当在流动相中添加DES-A、DES-C时（其中ChCl浓度均为80 mmol/L），其保留因子的变化与单独添加同等浓度ChCl基本一致（见表4）。这是因为它们均以ChCl为氢键受体，其中胆碱阳离子（Ch^+^）与固定相残留硅羟基负离子（Si-O^-^）间的离子交换作用是影响盐酸去甲替林保留因子的关键因素。然而，当流动相中添加氢键受体同为ChCl的DES-D后，盐酸去甲替林的保留因子略低于前者。这可能是由于其氢键给体甘油在反相体系中具有一定的溶剂洗脱能力，降低了碱性溶质的保留因子。而DES-B、DES-E采用疏水性较强的四甲基氯化铵、四乙基氯化铵作为氢键受体，它们与固定相疏水链结合较ChCl更紧密，对盐酸去甲替林与硅羟基间的次级相互作用屏蔽程度更高^［16］^，因此也会导致盐酸去甲替林保留偏弱。

**表4:** DESs作为添加剂对盐酸去甲替林和苯甲酸保留因子的影响（C18柱）

DES	*k*	
	Nortriptyline HCl^*^	Benzoic acid^**^
DES-A	3.18	3.14
DES-B	2.96	3.03
DES-C	3.17	>6.00
DES-D	3.01	3.17
DES-E	2.33	6.05
ChCl	3.12	3.23

* Mobile phase： ACN-13.8 mmol/L PB （pH 2.5， *C*_DES-A_=20.78 mg/mL， or *C*_DES-B_=23.66 mg/mL， or *C*_DES-C_=26.54 mg/mL， *C*_DES-D_=25.90 mg/mL， *C*_DES-E_=28.15 mg/mL， *C*_ChCl_ *=*11.17 mg/mL （*c*_ChCl_=80 mmol/L））（40∶60）. ** Mobile phase： ACN-9.2 mmol/L PB （pH 6.5， *C*_DES-A_=20.78 mg/mL， or *C*_DES-B_=23.66 mg/mL， or *C*_DES-C_=26.54 mg/mL， *C*_DES-D_=25.90 mg/mL， *C*_DES-E_=28.15 mg/mL， *C*_ChCl_ *=*11.17 mg/mL）（5∶95）.

与碱性化合物相比，DESs添加剂对酸性化合物保留行为的调控效果截然不同。如表4所示，经DES-E调控后，苯甲酸的保留因子显著高于其经其他DESs（DES-C除外）调控后的保留因子。这是因为当流动相pH高于苯甲酸的解离常数（p*K*_a_）时，DES-E中的四乙基氯化铵能与苯甲酸负离子形成疏水性较强的离子对。与之形成鲜明对比的是，经DES-C调控后苯甲酸的保留因子极高。这主要归因于：尽管缓冲液pH高达6.5，DES-C中的柠檬酸可将流动相的表观pH（^s^_w_pH）降低至2.35，使苯甲酸几乎完全以分子形态存在，从而显著增强了苯甲酸的保留。

综上，DESs氢键受体如ChCl提供的静电作用以及流动相^s^_w_pH均可影响酸、碱化合物在C18柱上的保留行为。因此，本工作以在流动相中添加DESs中常用氢键受体ChCl为例，结合流动相pH调控，系统探究不同类型化合物在C18柱上的分离规律，阐明了DESs调控化合物色谱行为的内在机制，并进一步探讨其在制备液相色谱及天然产物分离中的应用潜力。

### 2.2 以ChCl为添加剂对C18柱上碱分离效果的影响

#### 2.2.1 以ChCl为添加剂时碱在C18柱上的色谱行为

如上所述，在流动相中添加ChCl会导致碱性化合物（如盐酸去甲替林）与固定相间作用的变化，而pH对这种效果有着显著影响。如图1所示，在分析型色谱条件下（上样量0.5 μg），随着缓冲液pH从2.5增加至6.5，盐酸去甲替林保留逐渐增加。这是因为pH增加会导致硅羟基解离成Si-O^-^，其与盐酸去甲替林阳离子间的离子交换作用增强^［17］^。相比较而言，在流动相中添加ChCl后，盐酸去甲替林保留增加幅度较小。这是因为，ChCl解离出的Ch^+^会与盐酸去甲替林阳离子竞争结合Si-O^-^，削弱了这种离子交换作用。当缓冲液pH为2.5且流动相中添加ChCl时，盐酸去甲替林在C18柱上的保留比未添加时更高。这是因为在酸性条件下，硅羟基的解离被充分抑制。此时，ChCl中Cl^-^可与盐酸去甲替林阳离子形成中性离子对，消除了吸附于固定相表面的去甲替林阳离子间的静电排斥，从而增强了保留^［18］^。

**图1:**
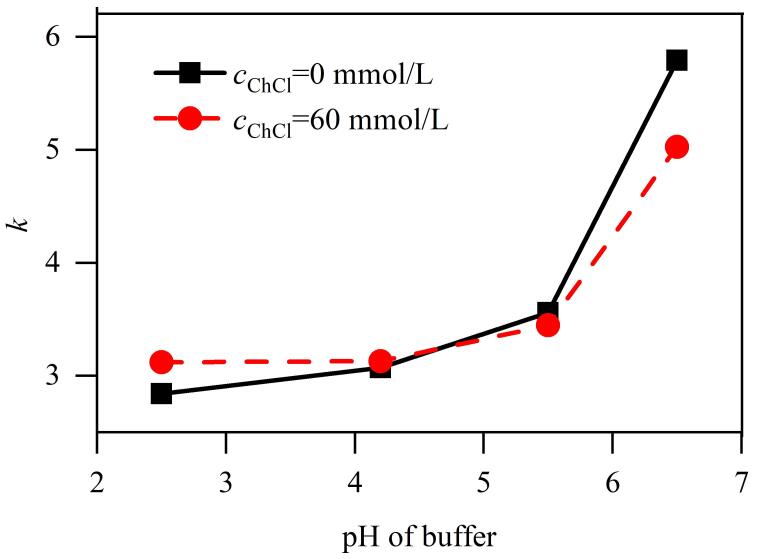
不同pH下在流动相中添加ChCl对盐酸去甲替林在C18色谱柱上保留因子的影响

盐酸去甲替林在C18柱上的过载行为进一步佐证了上述作用机制。如图2a所示，当缓冲液pH为2.5时，盐酸去甲替林在过载条件下的峰宽随流动相中ChCl浓度的增加逐渐变窄。在流动相中添加KCl后，峰宽也呈现相似的变化趋势（图2b），但同浓度下的峰宽显著大于添加ChCl时的峰宽。这说明：（1）即使在酸性环境中，仍有少量Si-O⁻存在；（2）添加ChCl对碱性化合物过载峰形的优化作用主要源于对这些Si-O^-^的屏蔽。从峰形特征分析，添加ChCl条件下，盐酸去甲替林过载峰的尾部陡峭程度高于添加KCl时，说明固定相上位点的均匀程度更好^［19］^，这有利于天然产物中生物碱类化合物的制备分离。

**图2:**
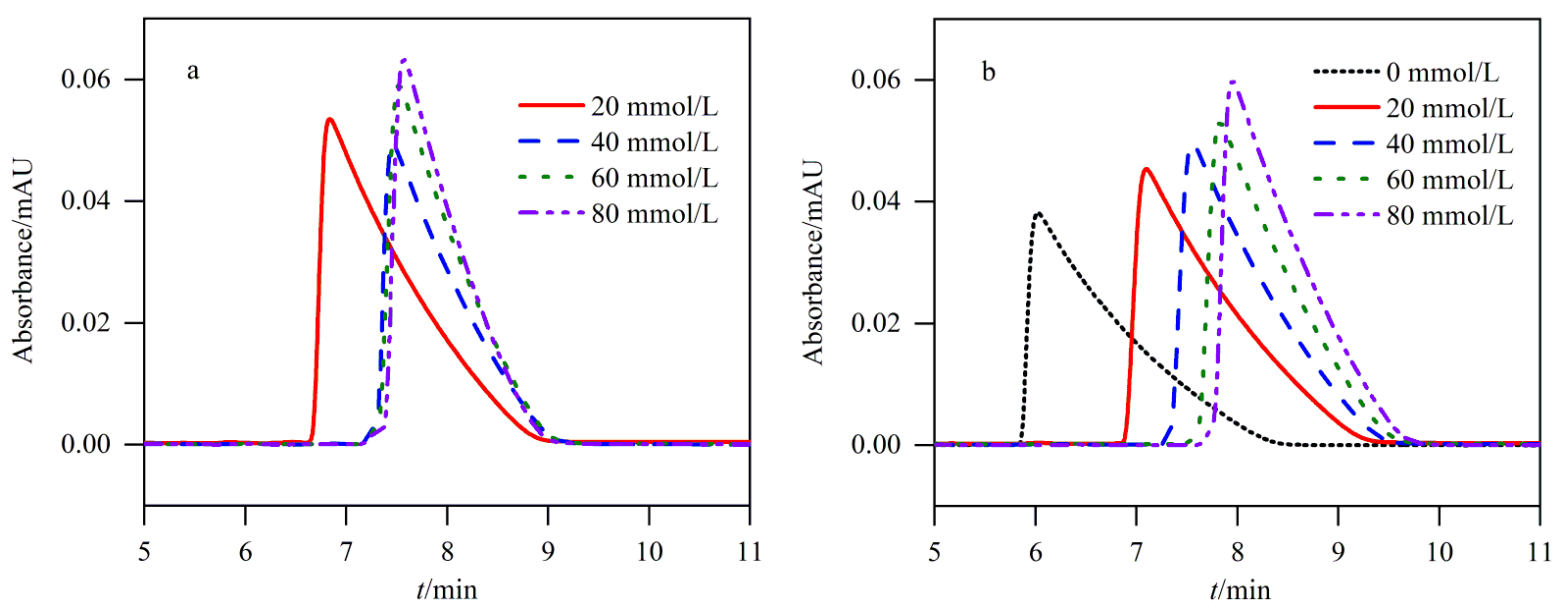
（a）氯化胆碱和（b）氯化钾添加浓度对盐酸去甲替林在分析柱上过载行为的影响

附图S1（https://www.chrom-China.com）展示了极微量进样（0.025 μg）条件下，添加ChCl对盐酸去甲替林色谱行为的影响。可以看出，在流动相中添加ChCl可显著缩短盐酸去甲替林的保留时间并降低峰宽。该现象表明，盐酸去甲替林色谱峰形的改善效果主要源自ChCl的添加，而非高上样量条件下造成的非线性吸附行为。

附图S2（https://www.chrom-China.com）展示了在流动相中添加含80 mmol/L ChCl的DES-A、DES-C和DES-D条件下，盐酸去甲替林在分析柱上的过载色谱图。与图2a相比，在添加DES-A和DES-C的条件下，盐酸去甲替林所呈现的峰宽和保留时间与添加ChCl高度相似，进一步印证了HBA是DES影响色谱行为主要因素。同时，在添加DES-D的条件下，盐酸去甲替林的保留时间略有缩短，过载峰宽也略有收窄，这可能与其氢键给体甘油具有一定洗脱能力有关。

#### 2.2.2 以ChCl为添加剂分离黄连提取物中的小檗碱

为验证上述结论在天然碱性化合物分离中的应用价值，以黄连提取物中的小檗碱为分离对象，开展载样量研究和半制备色谱纯化实验。如图3a和图3b所示，与盐酸去甲替林过载分离实验中观察到的规律类似，在酸性流动相（缓冲液pH 2.5）中添加ChCl在一定程度上压缩了小檗碱的峰宽（无论是否过载）。当缓冲盐pH升至6.5时，硅羟基趋向于完全解离，产生的Si-O^-^与小檗碱阳离子间的离子交换作用大幅增强，导致严重的拖尾和保留时间延长（图3c）。据此推测，在该条件下向流动相中添加ChCl可比酸性条件下更高程度地改善小檗碱的过载峰形。

**图3:**
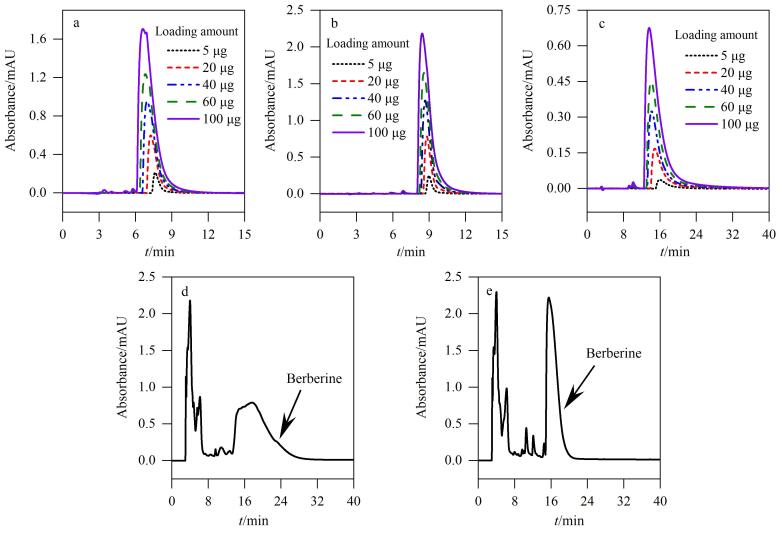
（a~c）流动相中添加ChCl时盐酸小檗碱在分析柱上的色谱图；黄连提取物在流动相中ChCl添加（d）前和（e）后在半制备柱上的色谱图

图3d和3e对比展示了在流动相（缓冲液pH 6.5）中添加ChCl对黄连提取物在半制备C18柱上分离效果的影响。当上样量高达10 mg时，经ChCl调控后，小檗碱的峰拖尾程度大幅降低，其与周围杂质的分离度得到了明显提升。该结果充分表明DESs在改善天然碱性化合物反相制备分离方面具备应用潜力。

### 2.3 以ChCl为添加剂对C18柱上酸分离效果的影响

#### 2.3.1 ChCl对C18柱上酸色谱行为的影响机制

对于酸性化合物，ChCl作为流动相添加剂对反相色谱分离效果的影响与pH值高度相关。如图4a所示，当流动相pH远低于苯甲酸p*K*_a_（4.20）时，苯甲酸的过载峰形符合Langmuir型吸附特征，而ChCl的添加对其峰形与保留无显著影响。原因在于，苯甲酸的解离几乎完全被抑制，而分子态的苯甲酸无法与ChCl中的Ch^+^产生静电相互作用。因此，苯甲酸在C18上的吸附主要来自其与疏水键合相间的疏水作用。而ChCl作为离子型化合物，对疏水作用的干扰很小。当缓冲液的pH升高至4.2和5.5时，苯甲酸部分解离，其色谱峰形符合反-Langmuir型吸附特征（图4b和4c）。

**图4:**
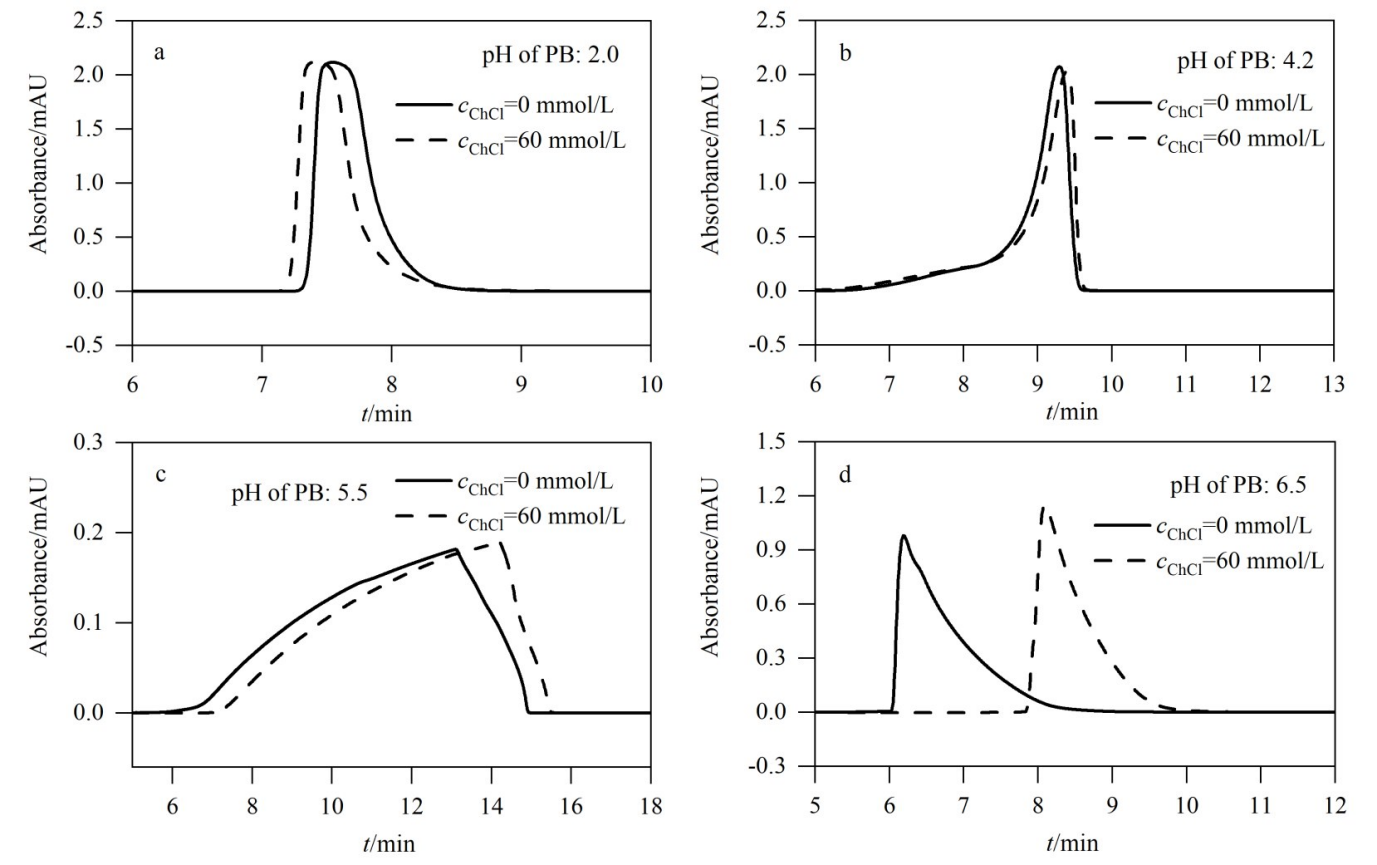
不同pH条件下在流动相中添加ChCl对苯甲酸在分析柱上过载行为的影响

与此同时，随着pH的升高，解离出的苯甲酸负离子可与流动相中的Ch^+^作用形成中性离子对，导致其在疏水固定相上的保留时间延长。苯甲酸解离程度越高，所形成的离子对比例越高，ChCl对苯甲酸在C18上保留的改变效果越明显。当缓冲液pH进一步升至6.5，苯甲酸趋向于完全解离，其过载峰形重新符合Langmuir型吸附特征^［19］^。该条件下，在流动相中添加ChCl可窄化苯甲酸的色谱峰宽（图4d），这归因于Ch^+^与苯甲酸负离子形成离子对后，降低了其在固定相表面的相互排斥^［13］^，从而增强了吸附容量。

除流动相pH外，酸性化合物自身的解离也影响分离效果^［19］^。酸性化合物解离出的H^+^可以降低流动相pH，进而通过影响自身解离状态来改变其吸附特征。附表S1显示在缓冲液pH为6.5的流动相中，随着苯甲酸浓度的增加，流动相的^s^_w_pH持续降低，进而抑制了苯甲酸的解离，导致其分子态比例相应增高。据此预测，随着上样量的增加，苯甲酸的吸附特征将由Langmuir型逐渐转向S型。图5a中不同上样量下苯甲酸峰形的变化证实了这一观点。当上样量低于50 μg时，苯甲酸的峰宽随上样量增加而增长迅速，表明吸附位点数量少，这不利于制备分离。当上样量为500 μg时，苯甲酸过载峰形开始呈现“前伸”现象^［20］^，符合S型吸附特征。

**图5:**
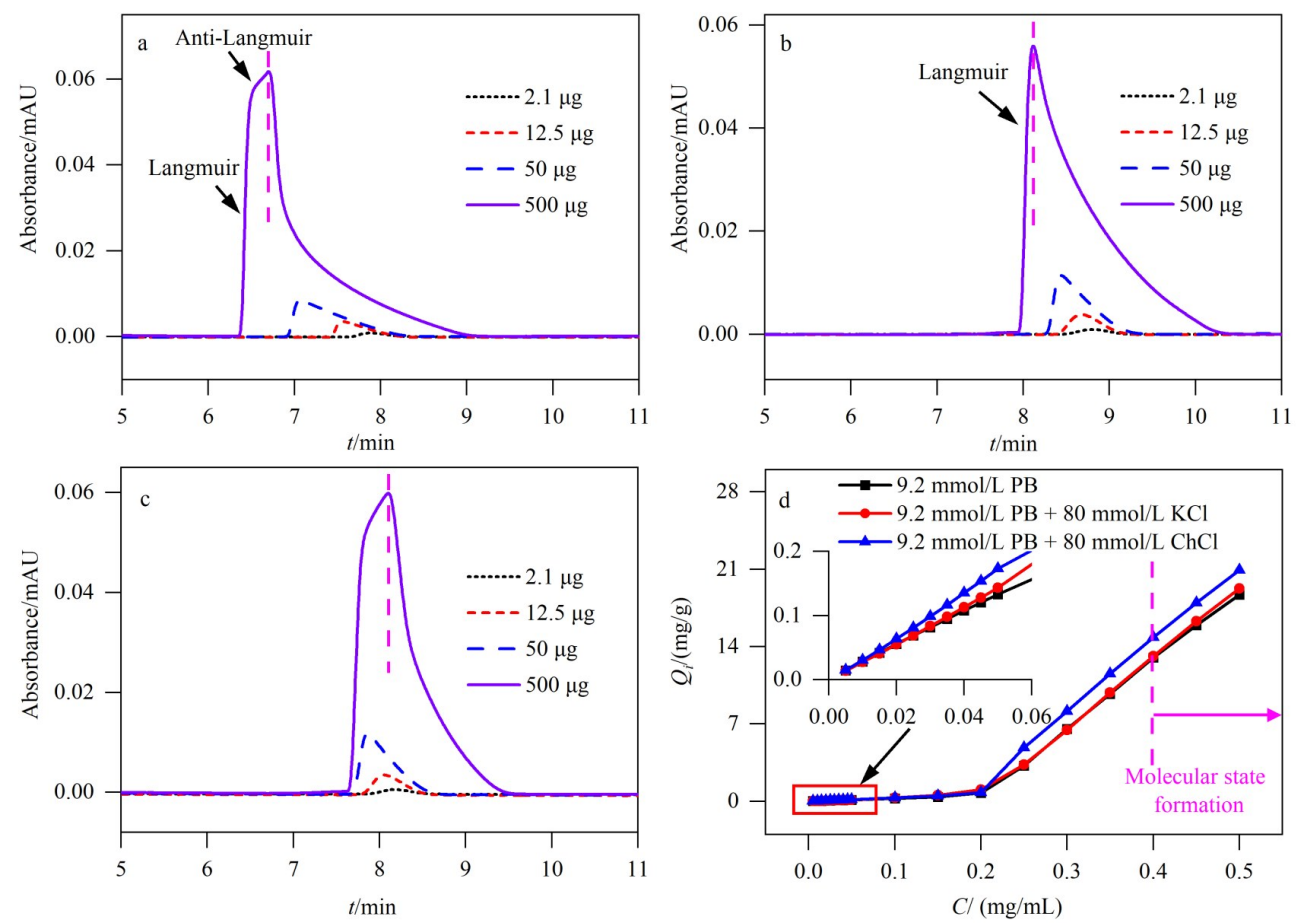
流动相添加剂对苯甲酸在分析柱上的过载行为影响

在流动相中添加ChCl可显著改善酸性化合物的过载分离效果。如图5b所示，当缓冲液中ChCl的浓度为80 mmol/L时，即使上样量达500 μg，苯甲酸过载峰形仍较好地符合Langmuir型吸附，表明吸附容量显著提升。Ch^+^与苯甲酸负离子间的静电作用被认为是导致该结果的主要因素。在流动相中添加相同浓度的KCl也能起到类似峰宽窄化作用，但苯甲酸过载峰形则呈现出明显的S型吸附（图5c），这可能与含KCl的流动相的^s^_w_pH较含ChCl的流动相偏低有关（见附表S1）。图5d中呈现的吸附等温线支持了上述解释。

ChCl的调控效果与缓冲盐浓度相关。如图6a所示，当PB浓度为18.2 mmol/L时，在流动相中添加ChCl后，苯甲酸的吸附等温线特征由未添加时的S型转为线性。这是因为随着缓冲盐浓度的增加，苯甲酸自解离对流动相pH的扰动减弱。当苯甲酸的质量浓度高于0.3 mg/mL时，苯甲酸在未经ChCl调控的C18柱上仍呈现S型吸附。

**图6:**
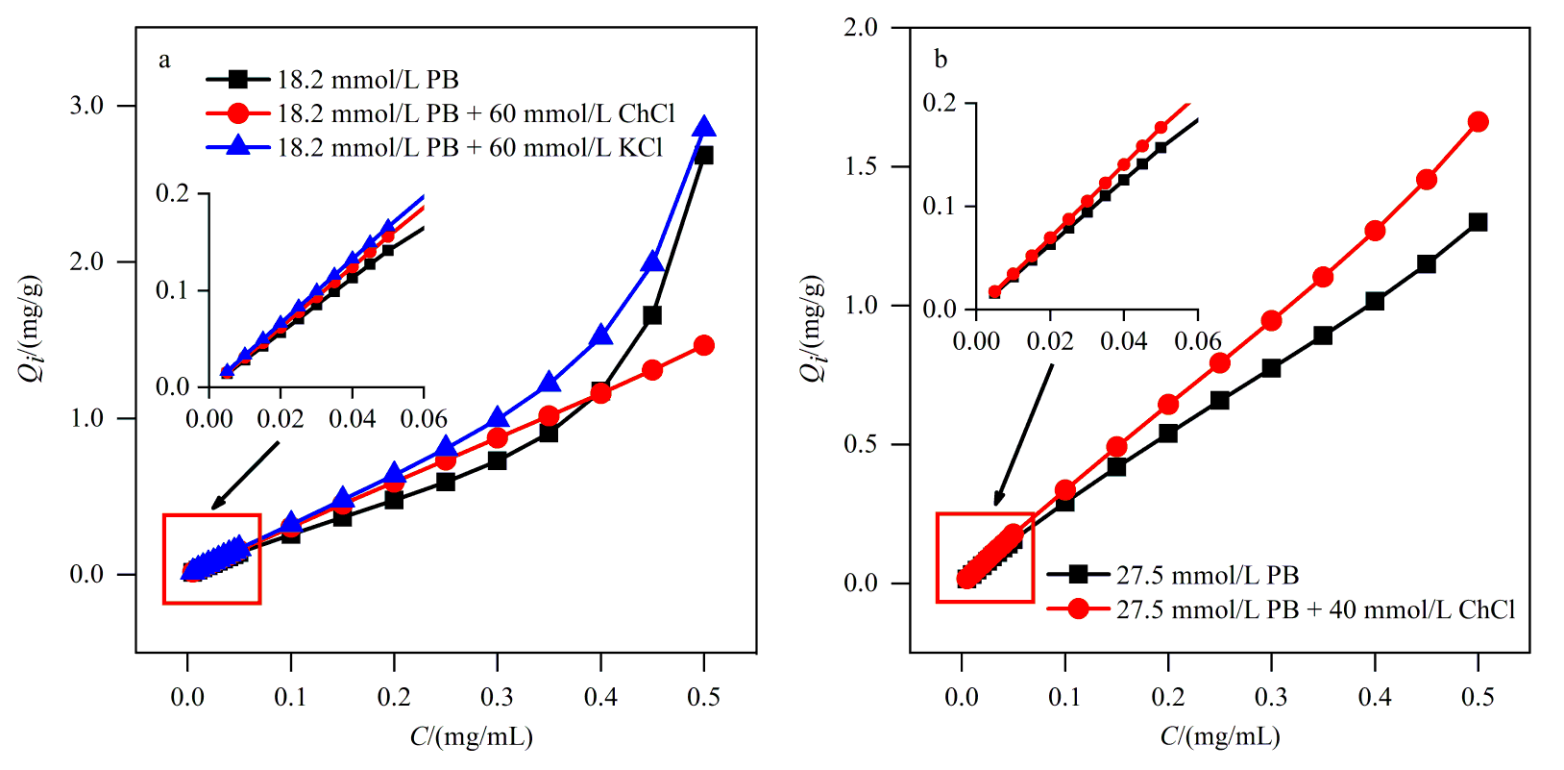
不同色谱条件下氯化胆碱对苯甲酸在分析柱上吸附行为的影响

与之不同的是，当流动相中添加ChCl后，即使质量浓度高达0.5 mg/mL，苯甲酸的吸附特征仍为线性。由于ChCl的添加对流动相的pH几乎无影响（附表S1），推测这种改变可能与ChCl对苯甲酸吸附过程中存在的溶质-溶质相互作用的抑制有关^［19］^。图6a还对比了在流动相中添加同等浓度KCl和ChCl后，苯甲酸在C18柱上的吸附等温线。在这种条件下，流动相的离子强度和Cl^-^浓度均与添加ChCl相同，Ch^+^和K^+^对苯甲酸吸附的调控效果因而能被清楚地区分。结果显示，虽然在流动相中添加KCl能够提高苯甲酸的吸附容量，其吸附特征仍呈S型，表明KCl对苯甲酸的吸附特征调控能力有限。这可能亦与KCl溶液的pH偏低有关（如附表S1所列）。当缓冲盐浓度进一步升至27.5 mmol/L时，流动相的pH几乎不随苯甲酸浓度变化而波动。在此条件下，无论流动相中是否添加ChCl，苯甲酸的吸附特征均呈线性。此时，苯甲酸吸附容量的提升主要取决于Ch^+^和苯甲酸负离子间的离子对效应（图6b）。

为验证ChCl对溶质-溶质相互作用的抑制作用，本研究考察了苯甲酸钾在含中性PB（pH 7.0）流动相条件下的过载峰形，避免了吸附过程中pH的波动。在流动相中无添加剂的条件下，苯甲酸钾过载峰形呈现了多位点吸附特征（图7a）。当流动相中添加KCl或ChCl后，过载峰洗脱后沿拐点出现时间较未添加时有所延迟（图7b、7c）。其中经ChCl调控后的过载峰洗脱后沿拐点滞后最显著、拖尾程度最低。然而，ChCl并未完全消除溶质-溶质相互作用，仅延缓其发生的进程。这种特征在制备色谱中具有重要应用价值：既可借助离子对效应增强吸附，又能在高上样量下借助溶质-溶质相互作用压缩峰宽，最终达到较高的制备分离效果。

**图7:**
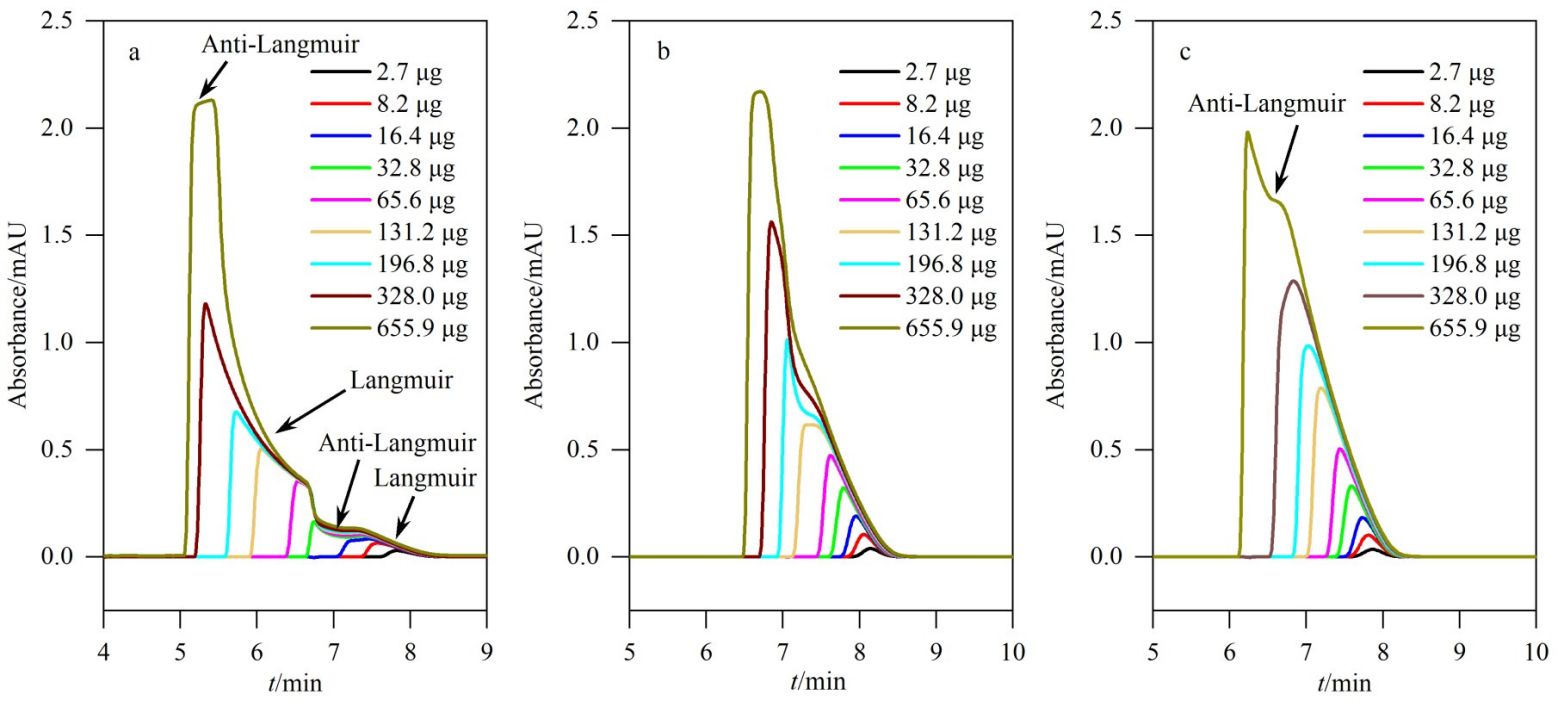
不同流动相条件下苯甲酸钾在分析柱上的过载效果图

#### 2.3.2 以ChCl为添加剂分离金银花提取物中的绿原酸

本工作以金银花提取物中绿原酸的分离为例，考察了在流动相中添加ChCl对天然酸性化合物的分离效果。与前述苯甲酸钾的色谱行为规律类似，流动相中的ChCl导致了绿原酸钾过载峰宽压缩和保留时间的延长。如图8a和8b所示，在流动相中ChCl的影响下，绿原酸钾的吸附特征从S型（图8a）转变为Langmuir型（图8b），表明ChCl的存在抑制了溶质-溶质相互作用。金银花提取物的半制备分离色谱结果进一步验证了该规律应用的可行性：在流动相中添加ChCl 后，绿原酸钾峰宽较未添加时更低，使得其与周围杂质的分离度显著增加，如图8c和图8d所示。

**图8:**
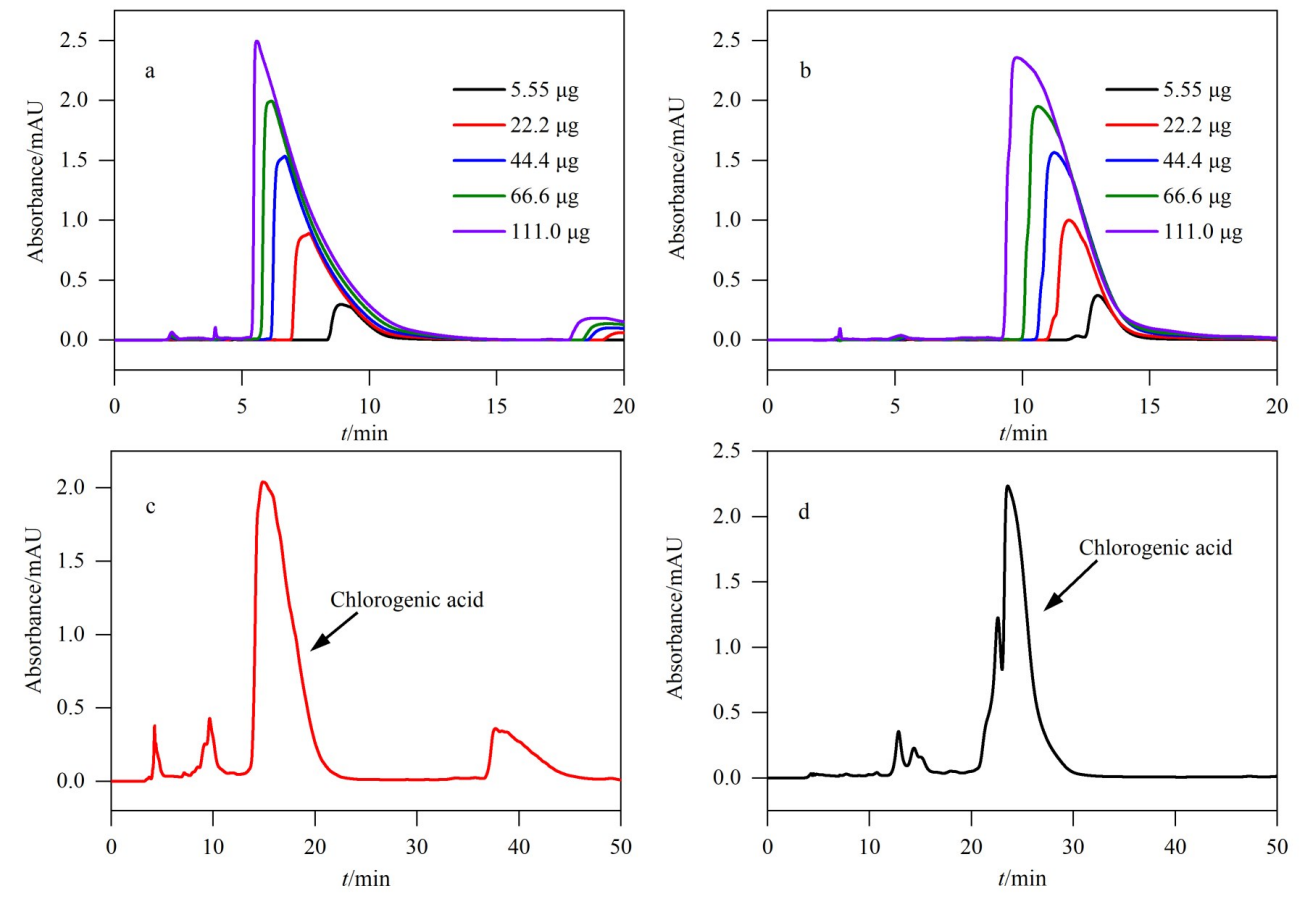
绿原酸钾在（a）未经改性和（b）经ChCl改性分析柱上的过载行为；（c）未经ChCl改性（d）经改性的半制备柱纯化金银花提取物

### 2.4 ChCl对非离子型化合物分离效果的影响——以山茱萸提取物中的环烯醚萜苷为例

作为典型天然非离子型化合物，环烯醚萜苷的解离能力极弱，其与固定相、添加剂或缓冲盐之间几乎无法产生静电作用，理论上ChCl对其分离无调控效果。通过图9a和9b中莫诺苷（环烯醚萜苷中的一种）的过载峰形对比可知，在流动相中添加ChCl对其过载峰形及保留时间几乎无影响。然而，山茱萸提取物的半制备分离实验结果（图9c和9d）表明，虽然莫诺苷的保留时间不受ChCl添加的影响，但莫诺苷与周围杂质的分离度稍有差异。为研究这种分离变化是否由ChCl引起，本研究按照图9c的色谱条件，收集制备了含有莫诺苷的组分，重新进行色谱分析。结果显示，收集到的组分中的莫诺苷的保留时间及峰形无显著改变，而某些杂质（如附图S3中的杂质1和杂质2）在C18柱上的保留行为受流动相中ChCl的显著影响。考虑到天然产物中往往含有很多可离子化化合物，尽管非离子型化合物自身的色谱行为不受ChCl影响，但ChCl仍能通过与非离子型化合物共洗脱的可离子化杂质产生静电作用，间接改变分离效果。

**图9:**
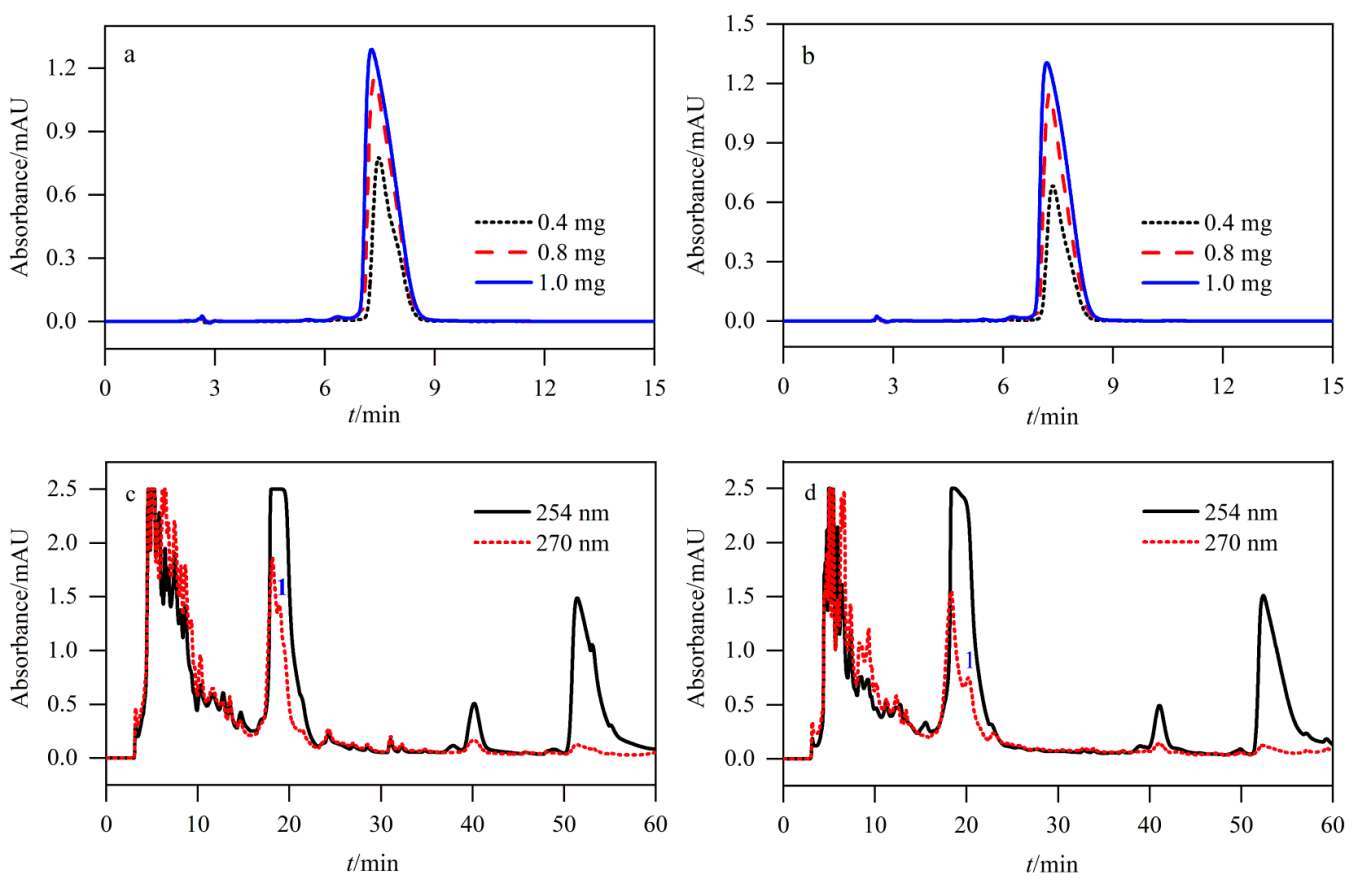
莫诺苷在（a）未经、（b）经ChCl改性的分析柱上的过载行为；山茱萸提取物在（c）未经、（d）经ChCl改性的半制备柱上的纯化

## 3 结论

本工作系统研究了DESs调控反相色谱的过载分离效果与机制，并分别通过黄连、金银花和山茱萸提取物的半制备分离，验证了其在天然化合物分离中的应用潜力。研究表明，在一定的pH与缓冲盐浓度下，DESs中的HBA能通过调节静电相互作用力或溶质-溶质相互作用，改变可离子化化合物在固定相上的保留行为和过载容量。与传统调pH等方式相比，这种调节方法较为温和，可在几乎不影响洗脱顺序的前提下实现分辨率优化，因此能作为反相制备色谱方法优化的有效补充策略。此外，中性化合物的分离纯化效果也可能通过DESs与可离子化杂质之间的静电相互作用得到改善，确保了该调节方法的广泛适用性。
